# *BrrA02.LMI1* Encodes a Homeobox Protein That Affects Leaf Margin Development in *Brassica rapa*

**DOI:** 10.3390/ijms241814205

**Published:** 2023-09-18

**Authors:** Pan Li, Yudi Wu, Xiangyang Han, Hui Li, Limin Wang, Bin Chen, Shuancang Yu, Zheng Wang

**Affiliations:** 1 State Key Laboratory of Vegetable Biobreeding, Beijing Vegetable Research Center, Beijing Academy of Agriculture and Forestry Science, Beijing 100097, China; lipan@nercv.org (P.L.); wuyudi@nercv.org (Y.W.); hanxiangyang@nercv.org (X.H.); lihui@nercv.org (H.L.); wanglimin@nercv.org (L.W.); wyj20101212@163.com (B.C.); yushuancang@nercv.org (S.Y.); 2 National Engineering Research Center for Vegetables, Beijing Vegetable Research Center, Beijing Academy of Agriculture and Forestry Science, Beijing 100097, China; 3 Beijing Key Laboratory of Vegetable Germplasms Improvement, Beijing 100097, China; 4 Key Laboratory of Biology and Genetics Improvement of Horticultural Crops (North China), Beijing 100097, China

**Keywords:** *Brassica rapa*, *LMI1-like*, leaf lobe, transformation, functional identification

## Abstract

Leaf margin morphology is an important quality trait affecting the commodity and environmental adaptability of crops. *Brassica rapa* is an ideal research material for exploring the molecular mechanisms underlying leaf lobe development. Here, we identified *BrrA02.LMI1* to be a promising gene underlying the QTL *qBrrLLA02* controlling leaf lobe formation in *B. rapa,* which was detected in our previous study. Sequence comparison analysis showed that the promoter divergences were the most obvious variations of *BrrA02.LMI1* between parental lines. The higher expression level and promoter activity of *BrrA02.LMI1* in the lobe-leafed parent indicated that promoter variations of *BrrA02.LMI1* were responsible for elevating expression and ultimately causing different allele effects. Histochemical GUS staining indicated that *BrrA02.LMI1* is mainly expressed at the leaf margin, with the highest expression at the tip of each lobe. Subcellular localization results showed that BrrA02.LMI1 was in the nucleus. The ectopic expression of *BrrA02.LMI1* in *A. thaliana* resulted in a deep leaf lobe in the wild-type plants, and lobed leaf formation was disturbed in *BrrA02.LMI11*-downregulated plants. Our findings revealed that *BrrA02.LMI1* plays a vital role in regulating the formation of lobed leaves, providing a theoretical basis for the selection and breeding of leaf-shape-diverse varieties of *B. rapa*.

## 1. Introduction

Leaves are essential organs that produce energy through photosynthesis in higher plants, playing an essential role in nutrient accumulation, gas exchange, light absorption, and water transport. Leaves are also the main edible organs of leafy vegetables and provide rich nutrients such as vitamins, soluble fiber, and mineral nutrition for humans. Leaves evolve different morphologies in the process of adapting to complex environments, which leads to functional variation in leaves [1]. Leaf morphology is mainly determined by the outline of the leaf margin, with the leaf margin categorized as smooth, serrated, or lobed [2,3,4]. Plants with deep leaf lobes have shown better adaptability to environmental stresses, such as drought, heat, and diseases, due to optimal canopy architecture [4].

Leaves originate from a mass of pluripotent cells at the flanks of the shoot apical meristem (SAM) [5]. The process of leaf morphogenesis involves three stages: leaf initiation (I), primary morphogenesis (PM), and expansion and secondary morphogenesis (SM) [6]. The leaf primordium forms via the lateral differentiation of cells from the peripheral region of the SAM. During the primary morphogenesis process, the basic form of the leaf, including leaf symmetry and the major subregions, is established [7]. The expansion and secondary morphogenesis stage—during which the leaf surface area and volume increase a thousandfold through cell division and differentiation, accompanied by the appearance of typical characteristics of mature leaves such as stomata and epidermal hairs—is much longer than the primary morphogenesis stage.

Leaf development and morphogenesis require extremely complex physiological and biochemical processes and involve crosstalk among transcription factors, miRNAs, and hormones [8,9,10,11]. The Class I KNOTTED1-LIKE HOMEOBOX (KNOX) family is an important transcription factor family that maintains meristem activity. In *Arabidopsis*, four members of the KNOX I family have been detected, *SHOOTMERISTEMLESS* (*STM*), *BREVIPEDICELLUS* (*BP*), *KNOTTED1-LIKE2* (*KNAT2*), and *KNOTTED1-LIKE6* (*KNAT6*), and the overexpression of either member leads to the appearance of lobed leaves [12]. The DRM1/ARP (dormancy-associated protein 1/auxin-repressed protein) family is highly conserved in different species and antagonizes KNOXI family gene expression during leaf development. *ASYMMETRIC LEAVES1* (*AS1*) and *AS2* form a repressor complex that directly binds to specific promoter regions of the KNOXI family and represses *KNOXI* gene expression [13,14]. *CUP-SHAPED COTYLEDON* (*CUC*) genes are expressed at the boundary of the leaves [15]. The functional loss of *CUC* genes has been found to lead to less dissected leaves in many species, including *Arabidopsis*, tomato, pea, potato, *Cardamine,* and *Aquilegia* [16,17]. *CUC2* was found to be targeted and negatively regulated by *miR164A*, and the functional loss of *miR164A* has been shown to lead to increased leaf serrations in *Arabidopsis*. In addition, negative feedback loops involving *PIN1* and *CUC2* result in regular leaf serration in *Arabidopsis* [18]. The overexpression of *LEAFY* (*LFY*) results in a deepened leaf lobe and complex leaf morphology in *Cardamine hirsuta* [19]. The HD-Zip I transcription factor, *LATE MERISTEM IDENTITY1* (*LMI1*), was first identified as a meristem identity regulator in *Arabidopsis*. *LMI1* and its homologs in many other species have been identified as influencing leaf morphogenesis [20,21]. The *REDUCED COMPLEXITY* (*RCO*) gene was found to be responsible for leaflet formation in *C. hirsuta* [20]. Class II TEOSINTE BRANCHED1, CYCLOIDEA, and PRO-LIFERATING CELL FACTORS (TCP) genes have been reported to regulate leaf morphology by preventing cell proliferation [22,23]. In addition to these gene families, some specific genes, including *SIMPLE LEAF3* (*SIL3*) [24], *BLADE-ON-PETIOLE1* (*BOP1*) and *BOP2* [25,26], *SAWTOOTH 1* (*SAW1*) and *SAW2* [27], *Squamosa-Promoter binding-like* (*SPL*) [28,29], and *JAGGED* (*JAG*) [30,31], have been reported to be involved in leaf morphology.

*Brassica* species are rich in leaf margin morphology diversity and provide abundant genetic resources and ideal materials for exploring the molecular mechanism of leaf development. In *B. napus*, two tandemly duplicated *LMI1-like* genes, *BnA10.LMI1* and *BnA10.RCO*, were reported to positively regulate lobed leaf formation [32,33]. In *Brassica oleracea*, *BoLMI1a*, a homolog of *LMI1*, was also predicted as the candidate gene regulating leaf lobe formation. In addition, Feng et al. [34,35] demonstrated that *BoALG10*, which encodes a glycosyltransferase, plays a vital role in leaf lobe development. In *Brassica rapa*, the physical interval at the distal end of chromosome A10, which is composed of a rich quantitative trait locus (QTL) of the lobed leaf trait, is a research hotspot. However, the molecular mechanisms underlying leaf lobe development are poorly understood. In our previous studies, two regions related to leaf lobe formation, *qBrrLLA10* and *qBrrLLA02*, were identified with bulked segregant analysis sequencing (BSA-seq) using a segregation population originating from the deeply lobed-leaf line MM and the serrated leaf line BY. We have already verified that *BrrRCO* was the causal gene underlying the *qBrrLLA10* locus, which positively regulates lobed-leaf formation [36]. However, the regulatory mechanism of the *qBrrLLA02* locus in leaf lobe formation remains unknown. Here, we provide strong evidence that *BrrA02.LMI1* is the candidate gene underlying the *qBrrLLA02* locus, and *BrrA02.LMI1* positively regulates leaf lobe formation independent of *BrrRCO* expression, providing novel insights into the regulatory network of lobed leaf formation in *Brassica* crops.

## 2. Results

### 2.1. Candidate Gene Identification Underlying the qBrrLLA02 Locus

We have shown that two major QTLs, *qBrrLLA02* and *qBrrLLA10*, are responsible for lobed leaf formation in *B. rapa* based on BSA-seq using an F_2_ segregation population originating from the cross between a deeply lobed-leaf turnip inbred line MM and a serrated leaf Chinese cabbage inbred line BY. Our previous study indicated that *BrrRCO* was the causal gene underlying the *qBrrLLA10* locus, and the molecular mechanism of *BrrRCO* in regulating the formation of lobed leaves was clearly elucidated. Here, we further tested whether the *BraA02g001070.3C* gene is the candidate gene underlying the *qBrrLLA02* locus regulating leaf lobe development in *B. rapa*.

**Figure 1:**
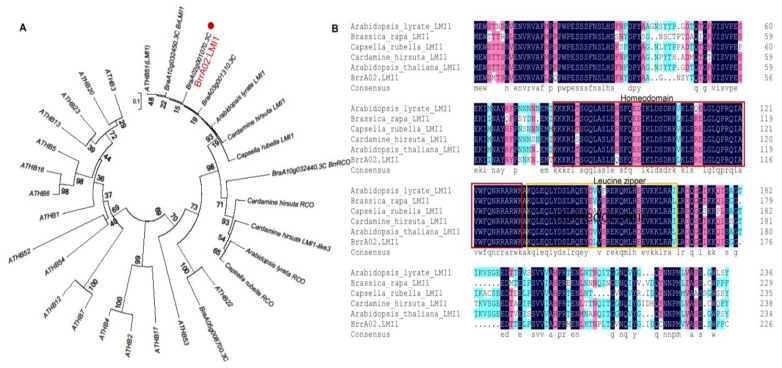
(**A**) Phylogenetic tree of BrrA02.LMI1 and LMI1-like proteins from various species. (**B**) Protein sequence alignment of LMI1 proteins from different species.

Among these annotated genes in the *qBrrLLA02* interval on chromosome A02, the gene *BraA02g001070.3C*, which was functionally annotated as associated with leaf morphogenesis, caught our attention. We constructed a phylogenetic tree using *BraA02g001070.3C* and its putative homeologs in *A. thaliana* and other species (Figure 1A), which indicated that *BraA02g001070.3C* is a homolog of *A. thaliana LMI1*, which is required for leaf serration and bract formation [37]; therefore, we renamed *BraA02g001070.3C* as *BrrA02.LMI1*. The conserved domain analysis revealed that BrrA02.LMI1 contains a typical homeobox domain and a leucine zipper domain (Figure 1B).

### 2.2. Genotyping Analysis of BrrA02.LMI1 in B. rapa

**Figure 2:**
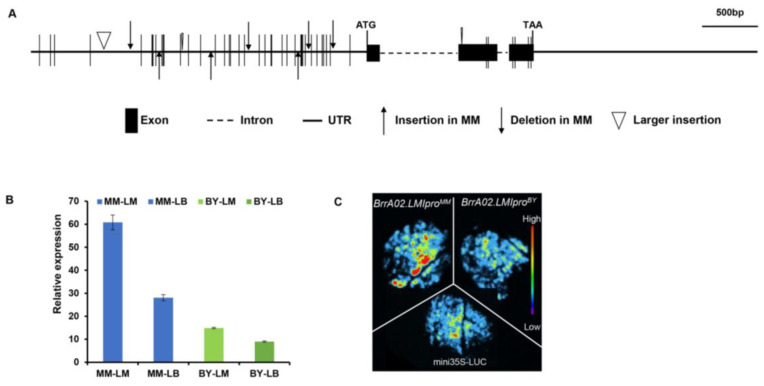
Gene structure, expression analysis, and promoter activity assays of *BrrA02.LMI1* in lobed-leaf MM and serrated leaf BY. (**A**) Schematic genetic variations of *BrrA02.LMI1* between the two parents. (**B**) Transcriptional expression of *BrrA02.LMI1* in leaf base and leaf margin from MM and BY. The *B. rapa GAPDH* gene was used as a reference gene to normalize the expression levels of *BrrA02.LMI1.* Data represent the mean ± SD (*n* = 3). (**C**) Promoter activity assays of *BrrA02.LMI1* in lobed-leaf MM and serrated leaf BY.

To further identify whether *BrrA02.LMI1* was the candidate gene underlying the *qBrrLLA02* locus, we conducted a genomic sequence alignment of *BrrA02.LMI1* between the two parental lines. We isolated and compared the genomic DNA segments, including the approximately 3 kb promoter sequence, gene body, and 2 kb 3′ flanking region of *BrrA02.LMI1* between MM and BY. *BrrA02.LMI1* encodes a protein comprising 227 amino acids. A 12 bp insertion and three nonsynonymous SNPs were detected in the coding sequence (Figure 2A and Figure S1A), which led to a four-amino-acid insertion and four-amino-acid substitution in the lobed-leaf MM compared with the corresponding positions of the serrated leaf BY (Figure S1B). In the promoter sequence, abundant polymorphisms, including 10 InDels and 28 SNPs, were identified between MM and BY (Figure 2A and Figure S1C). We then investigated the expression patterns of *BrrA02.LMI1* in parental lines. The fifth mature leaves of both parents were divided into two parts: leaf margin and leaf base, which were used for qRT-PCR assays. The results indicated that the *BrrA02.LMI1* expression in different leaf segments of MM was significantly higher than that in BY, with the highest expression at the leaf margin of MM (Figure 2B). Therefore, we suspected that variations in the promoter sequence play a vital role in determining the activity of *BrrA02.LMI1*. To address this question, the promoter activity of *BrrA02.LMI1* in MM and BY was detected using transient transcription activity assays. The luciferase assay showed that the promoter activity of the *BrrA02.LMI1* of deeply lobed-leaf MM was significantly higher than that of the serrated leaf BY (Figure 2C). In light of these results, we speculated that the promoter variations of *BrrA02.LMI1* may be responsible for increased expression levels and promoter activity, which induced the formation of leaf lobes in *B. rapa*. 

### 2.3. Tissue-Specific Expression Patterns of BrrA02.LMI1 in B. rapa

**Figure 3:**
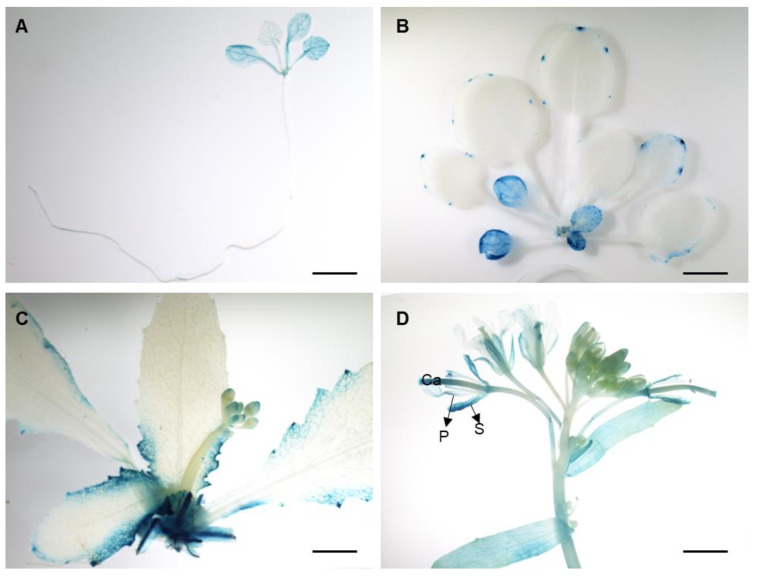
GUS histochemical staining assays in *proBrrA02.LMI1::GUS* transgenic *Arabidopsis*. Representative histochemical staining in (**A**) two-leaf stage seedlings, (**B**) slightly older seedlings, (**C**) bolting stage seedlings, and maturing flowers (**D**). S: sepals; P: petals; Ca: carpel. Scale bars: 500 μm.

To obtain the detailed tissue-specific expression patterns of *BrrA02.LMI1*, we constructed the vector *proBrrA02.LMI1::GUS*, fusing the promoter sequence of *BrrA02.LMI1*^MM^ to the GUS reporter gene and transformed it into wild-type *Arabidopsis*. The *BrrA02.LMI1::GUS* expression was observed in homozygous T3-positive transgenic lines. As shown in Figure 3A, *BrrA02.LMI1::GUS* expression was observed in all tissues during the two-leaf stage. In the slightly older seedling, *BrrA02.LMI1::GUS* expression was more visible in the proximal leaf margins, with the deepest stain in the serrations (Figure 3B). At the bolting stage, *BrrA02.LMI1* was expressed in the margins and serrations of bracts (Figure 3C). In addition, we observed GUS signals in the margins of sepals, petals, and carpels in maturing flowers (Figure 3D). These findings indicated that *BrrA02.LMI1* may be involved in the development of leaf margins and floral organs.

### 2.4. Subcellular Localization of BrrA02.LMI1

**Figure 4:**
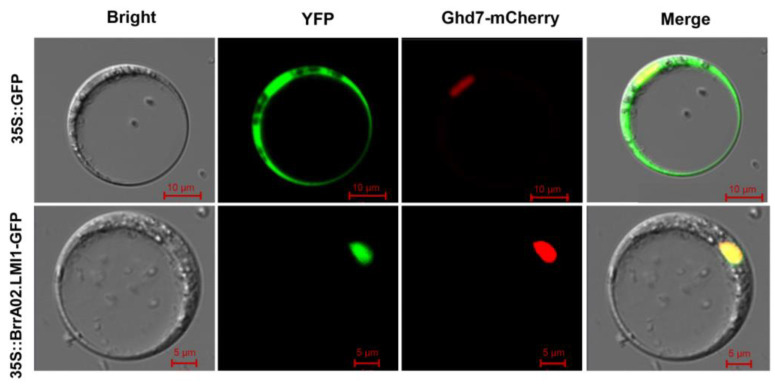
Subcellular localization of BrrA02.LMI1-GFP fusion protein in *B. rapa* protoplasts.

We constructed a *p35S:: BrrA02.LMI1-GFP* vector containing the *BrrA02.LMI1* coding sequence fused in frame with green fluorescent protein (GFP) under the control of the CaMV35S promoter to determine subcellular localization. Then, the recombinant and control plasmids were transferred into *B. rapa* protoplasts. As shown in Figure 4, the GFP signal of 35S:: BrrA02.LMI1-GFP was only visible in the nucleus, which is consistent with the function of BrrA02.LMI1 as transcription factors.

### 2.5. Function of BrrA02.LMI1 in Regulating Lobed Leaf Formation

**Figure 5:**
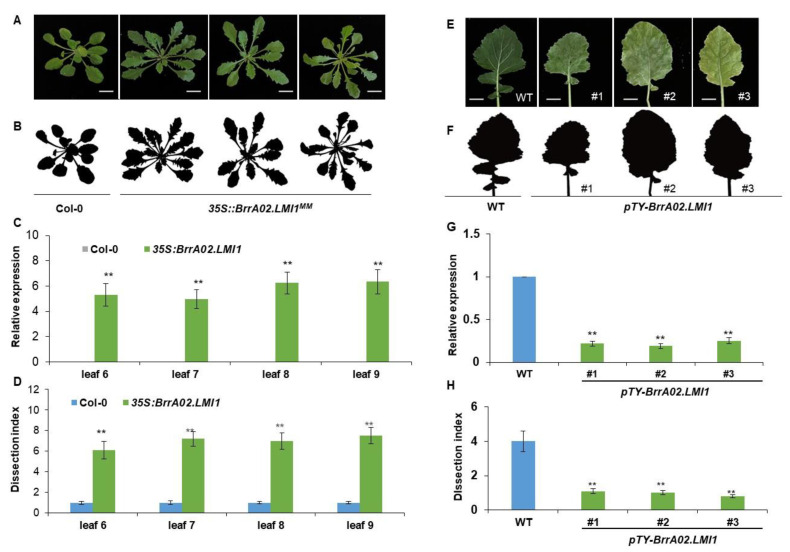
Phenotypic analysis of *BrrA02.LMI1* overexpression in *Arabidopsis* plants and *BrrA02.LMI1*-silenced MM plants. (**A**,**B**) *BrrA02.LMI1*-overexpressing *Arabidopsis* plants showed deeply lobed leaves. Bars = 1 cm; (**C**,**D**) *Arabidopsis* overexpression lines had a higher expression of *BrrA02.LMI1* and dissection index than that of the wild type. The *ACTIN2* (*At3g18780*) gene in *Arabidopsis* was used as reference genes to normalize the expression levels of *BrrA02.LMI1*. Three biological replicates were used for each PCR, and for each replicate, three leaves were sampled. Data represent the mean ± SD (*n* = 3). Double asterisk denotes a statistically significant difference to the Col-0 wild type in the Student’s *t*-test (*p* < 0.001). (**E**,**F**) *BrrA02.LMI1*-silenced plants showed a strongly reduced lobed-leaf phenotype. Bars = 1 cm; (**G**,**H**) *BrrA02.LMI1*-silenced plants had a downregulated expression of *BrrA02.LMI1* and reduced dissection index. Three biological replicates were used for each PCR, and for each replicate, three leaves were sampled. The *B. rapa GAPDH* gene was used as a reference gene to normalize the expression levels of *BrrA02.LMI1.* Data represent the mean ± SD (*n* = 3). Double asterisk denotes a statistically significant difference to the wild type in the Student’s *t*-test (*p* < 0.001).

To test the function of *BrrA02.LMI1* in lobed leaf formation, we transferred the constructs *35S::BrrA02.LMI1*^MM^ and *35S::BrrA02.LMI1*^BY^ overexpressing the coding sequence of the *BrrA02.LMI1* of lobed-leaf MM and serrated leaf BY into *Arabidopsis* Col-0 plants under the control of the *CaMV35S* promoter. Approximately 22 and 15 independent *35S::BrrA02.LMI1^MM^*- and *35S::BrrA02.LMI1^BY^*-positive transgenic lines were obtained after hygromycin B resistance selection. The *35S::BrrA02.LMI1^MM^* (Figure 5A,B) and *BrrA02.LMI1^BY^* (Figure S2A) lines both displayed a deep leaf lobe and an increased dissection index (Figure 5C and Figure S2B). The expression of *BrrA02.LMI1* in the overexpression lines was also significantly higher than that in the wild type (Figure 5D). These results demonstrated that the coding sequences of *BrrA02.LMI1* are most likely functionally equivalent in MM and BY.

We further confirmed *BrrA02.LMI1’s* function by using a turnip yellow mosaic virus (TYMV)-induced gene silencing (VIGS) method. The associated vector has been extensively used in *Arabidopsis* and *Brassica* species [38,39,40,41,42]. To construct a pTY-*BrrA02.LMI1* vector, the 80 -nt palindromic sequence specific to *BrrA02.LMI1* was synthesized and ligated to the pTY vector. The recombinant pTY-*BrrA02.LMI1* plasmids were used to inoculate seedlings of the lobed-leaf parent MM using a previously published method [36]. In MM plants treated with pTY-*BrrA02.LMI1*, the expression of *BrrA02.LMI1* was significantly downregulated in seedlings treated with pTY-*BrrA02.LMI1* (Figure 5G,H), and no obvious expression level alteration of the *BrrA02.LMI1* homologs, *BrLMI1* and *BrrRCO*, was detected (Figure S3A,B). In addition, leaf lobe formation in *BrrA02.LMI1*-silenced plants was disturbed, and the leaves became rounder compared with those of the controls (Figure 5E,F). These findings showed that *BrrA02.LMI1* plays a positive regulatory role in leaf lobe development in *B. rapa*.

## 3. Discussion

Leaves are important vegetative organs in plants, and leaf margin morphology is not only a reflection of plant diversity but also an adaptation of plants to the environment. Leaf lobe traits have many advantages in practice. In our previous study, in order to identify the candidate genes controlling lobed leaf traits in *Brassica rapa*, a genetic population was constructed using a turnip inbred line MM with lobed leaves and a Chinese cabbage inbred line BY with serrated leaves [36]. Two QTLs, *qBrrLLA10* and *qBrrLLA02*, were detected using this F_2_ population based on BSA-seq, and the molecular mechanism underlying the *qBrrLLA10* locus was clearly elucidated. However, little is known about the candidate genes and regulatory mechanism underlying *qBrrLLA02*. In the present study, an *LMI1* homolog from the European turnip line MM was identified as the causal gene underlying the *qBrrLLA02* locus and was named *BrrA02.LMI1*. The sequence variations between the two parents, the spatiotemporal expression patterns, and the function of *BrrA02.LMI1* were systematically analyzed, thereby helping to elucidate the genetic and molecular mechanisms underlying the involvement of the *BrrA02.LMI1* gene in leaf lobe formation in *B. rapa*.

Many sequence variations were detected in the *BrrA02.LMI1* promoter between the two parental lines. In addition, the expression level (Figure 2B) and promoter activity (Figure 2B) of *BrrA02.LMI1* in lobed-leaf MM was substantially higher than that in serrated leaf BY. Therefore, we speculated that the promoter variation of *BrrA02.LMI1* was responsible for different leaf margins in the two parents. The transgenic experimental results showed that the overexpression of the *BrrA02.LMI1* allele from MM and BY in *Arabidopsis* both led to increased leaf complexity (Figure 5A–D and Figure S2), which suggested that the function of the *BrrA02.LMI1* coding sequence is equivalent in MM and BY. Collectively, these findings indicated that the expression level of *BrrA02.LMI1* is positively related to the formation of leaf lobes in *B. rapa*, and that cis-regulatory divergences led to different leaf margin phenotypes between parental lines, which is consistent with previous reports in other species. In upland cotton, tandem repeat sequences in the *GhLMI1-D1b* promoter region enhance gene expression, leading to deep leaf lobes [21]. In *B. napus*, promoter variations of *BnA10.LMI1* determine the formation of lobed leaves in rapeseed [32,33]. In ornamental kale, the higher expression level of *BoLMI1* in the lobed-leaf parent was found to be due to numerous variations in the promoter region [26]. In zucchini, a few variations in the promoter led to stronger *CpDll* promoter activity in the deeply lobed-leaf parent than in the entire-leaf parent [43]. However, how promoter sequence variations affect gene expression and thus regulate the formation of leaf lobes still requires further investigation. To determine the mechanism of *BrrA02.LMI1* more completely, the upstream factors regulating the expression of the *BrrA02.LMI1* gene need to be further explored. In addition, the subcellular localization results showed that *BrrA02.LMI1* is in the nucleus, which is consistent with transcription factor patterns and suggests that *BrrA02.LMI1* may regulate leaf morphology by regulating the expression of downstream target genes.

The formation of both compound leaves and serrated leaf margins has a dosing effect [44]. In both simple-leaf and compound-leaf species, the appearance of serrations or leaflets depends on the time of their initiation. If primordial initiation occurs after leaflet unfolding and formation, then serrations will form instead of leaflets [45]. In *Cardamine hirsuta*, *ChLMI1* expression is limited to the leaf margin, while *RCO* is expressed at the base of the leaf, which is the location of vigorous cell division in the leaves, thus increasing the number of leaflets. The different expression positions of *ChLMI1* and *RCO* determine their respective biological processes during leaf development [20]. In our study, *BrrA02.LMI1* was mainly expressed at the margin of the leaf derived from the leaf primordium, and the expression region of *BrrA02.LMI1* determined its involvement in leaf lobe formation rather than in the establishment of compound leaf patterns.

As a growth repressor, *LMI1* participates not only in the formation of leaf serrations but also in the development of other plant organs. In *Arabidopsis*, *LMI1* is a meristem identity regulator and participates in bract formation and the transformation of stipules to leaves [37,46]. In pea, the homologous gene of *LMI1*, *Tl*, is expressed in tendrils, and the tendrils in *tl* mutants can be transformed into leaves [47]. In our study, some *BrrA02.LMI1* ectopic overexpression in *Arabidopsis* lines showed increased rosette leaves before bolting and more bracts in the reproductive stage (Figure S4), which underscores the idea that *LMI1* and *LMI1*-like genes are pleiotropic in regulating plant growth and development. In addition, other members of Class I HD-Zip genes have also been proven to participate in leaf development. *HAHB4* is a subclass I HD-Zip protein found in sunflower, and its overexpression in *Arabidopsis* transgenic plants showed shorter stems and internodes, rounder leaves, and enhanced drought resistance [48]. The overexpression of *ATHB13* can lead to abnormalities in the development of cotyledons and true leaves and incomplete connections between petioles and leaf sheaths [49]. *ATHB16*-overexpressing plants were found to display larger leaves and delayed flowering times [50]. Taken together with the findings of previous studies, our results revealed that the function of HD-Zip I genes involved in the formation of lobed leaves may be conserved across dicotyledons. However, it is necessary to investigate more HD-Zip I members and mine the endogenous and exogenous upstream factors that regulate the expression of HD-Zip I genes and downstream functional genes regulated by HD-Zip I genes. These results will provide a comprehensive understanding of the molecular mechanisms of this important gene family in regulating leaf lobe formation.

In conclusion, we identified *BrrA02.LMI1* as the causal gene underlying the *qBrrLLA02* locus and confirmed that *BrrA02.LMI1* plays a positive regulatory role in controlling leaf lobe formation in *B. rapa*. The results will further enrich the understanding of the molecular mechanism of leaf lobe formation, providing a theoretical basis to facilitate the breeding of leaf-shape-diverse varieties of *B. rapa* and other *Brassica* crops.

## 4. Materials and Methods

### 4.1. Plant Materials

A lobed-leaf line MM and a serrated leaf line BY, which were described in previous reports [36], were used in this study. Lobed-leaf MM and *Arabidopsis* thaliana Col-0 were used as transformation receptors for virus-induced gene silencing (VIGS) and ectopic overexpression assays. The leaf dissection index was calculated according to the formula (perimeter squared)/(4π × area) [18].

### 4.2. RNA Extraction and Expression Analysis of BrrA02.LMI1

RNA samples from various leaf segments of MM and BY were collected. RNA extraction, first-strand cDNA synthesis, and qRT-PCR were performed as described previously [36]. The *B. rapa GAPDH* [51] and *ACTIN2* (At3g18780) [52] genes in *Arabidopsis* were used as reference genes to normalize the expression level of the candidate gene, and the 2^−∆∆Ct^ method was used to calculate the relative expression of the candidate gene. Three biological replicates and technical replicates were used for each PCR.

### 4.3. Phylogenetic Analysis of BrrA02.LMI1

To construct the phylogenetic tree and perform the multiple sequence alignment analysis, the homologs of BrrA02.LMI1 were searched against the NCBI database, TAIR databases, and *Brassica* database using the BrrA02.LMI1 protein as a query. All deduced protein sequences were aligned using DNAMAN 9 software. MEGA software (MEGA 6.0) [53] was used to construct the phylogenetic tree with the neighbor-joining method. The bootstrap repetition value was 1000.

### 4.4. Subcellular Localization of BrrA02.LMI1

We amplified the open reading frame (without a termination codon) of *BrrA02.LMI1* in MM and fused them in frame with green fluorescent protein (GFP) to construct the *p35S::BrrA02.LMI1^MM^-GFP* vector under the control of the 35S promoter. The *p35S::BrrA02.LMI1MM-GFP* and *35S::GFP* control plasmids were then transformed into protoplasts of Chinese cabbage. We observed the GFP signals at 2 days after transfection using a laser-scanning confocal microscope (Zeiss LSM 710).

### 4.5. GUS Staining

To explore the tissue-specific expression patterns of *BrrA02.LMI1*, we cloned approximately 2 kb promoter sequences of *BrrA02.LMI1* and inserted them into the PacI/XbaI-linearized binary vector pMDC163. The recombinant plasmid was then transformed into *Arabidopsis* Col-0 plants. For GUS staining, homozygous T3 transgenic positive lines were developed. Seedlings at different stages were sampled and immersed in GUS staining buffer at the same time and then incubated at 37 °C overnight in the dark. The treated samples were successively decolorized with 75% (*v/v*) ethanol and observed using a Nikon microscope (SMZ1500).

### 4.6. Functional Analysis of BrrA02.LMI1

The full coding DNA sequence (CDS) of *BrrA02.LMI1* in deeply lobed-leaf MM and serrated leaf BY were amplified, and the purified products were inserted into the KpnI/SpeI-linearized binary vector *pCAMBIA1301* driven by the *CaMV 35S* promoter. The *35S::BrrA02.LMI1^MM^* and *35S::BrrA02.LMI1^BY^* were then transformed into wild-type *Arabidopsis,* as previously described [36]. Transgenic T1 plants were screened with Murashige and Skoog medium supplemented with 50 mg L^−1^ hygromycin, and homozygous T3 lines were used for further experiments.

The function of *BrrA02.LMI1* was further confirmed via knockout assays using a TYMV-based VIGS system. To construct VIGS vector *pTY-BrrA02.LMI1*, an 80-nt palindromic exon sequence specific to *BrrA02.LMI1* was synthesized and inserted into the SnaBI-linearized pTY vector with a T4 DNA ligase. The *pTY-BrrA02.LMI1* and *PTY* (negative control) plasmids were then introduced into *Escherichia coli Stb13* cells for plasmid extraction using the OMEGA Plasmid Giga Kit. The purified *pTY-BrrA02.LMI1* and *PTY* plasmids were diluted to 300–500 ng/µL and then used to infiltrate the second to the fourth fully expanded true leaves of MM at the four-leaf stage. For the *BrrA02.LMI1* function analysis, the leaf lobe phenotype was recorded and compared between treated and control plants. The silencing efficiency of *BrrA02.LMI1* in MM plants was determined using a Qrt–PCR.

All the primers used in this study were listed in Table S1.

## Data Availability

The data presented in this study are available on request from the corresponding author.

## Supplementary Materials

The supporting information can be downloaded at: https://www.mdpi.com/article/10.3390/ijms241814205/s1.
