# Supplementary material for: BrrA02.LMI1 Encodes a Homeobox Protein That Affects Leaf Margin Development in Brassica rapa
Source: Int J Mol Sci. 2023 Sep 18;24(18):14205. doi: 10.3390/ijms241814205 (PMC10532282; doi:10.3390/ijms241814205)
Supplement: Supplementary file 1 [file ijms-24-14205-s001.zip › Supplementary Figures-20230721.pdf]

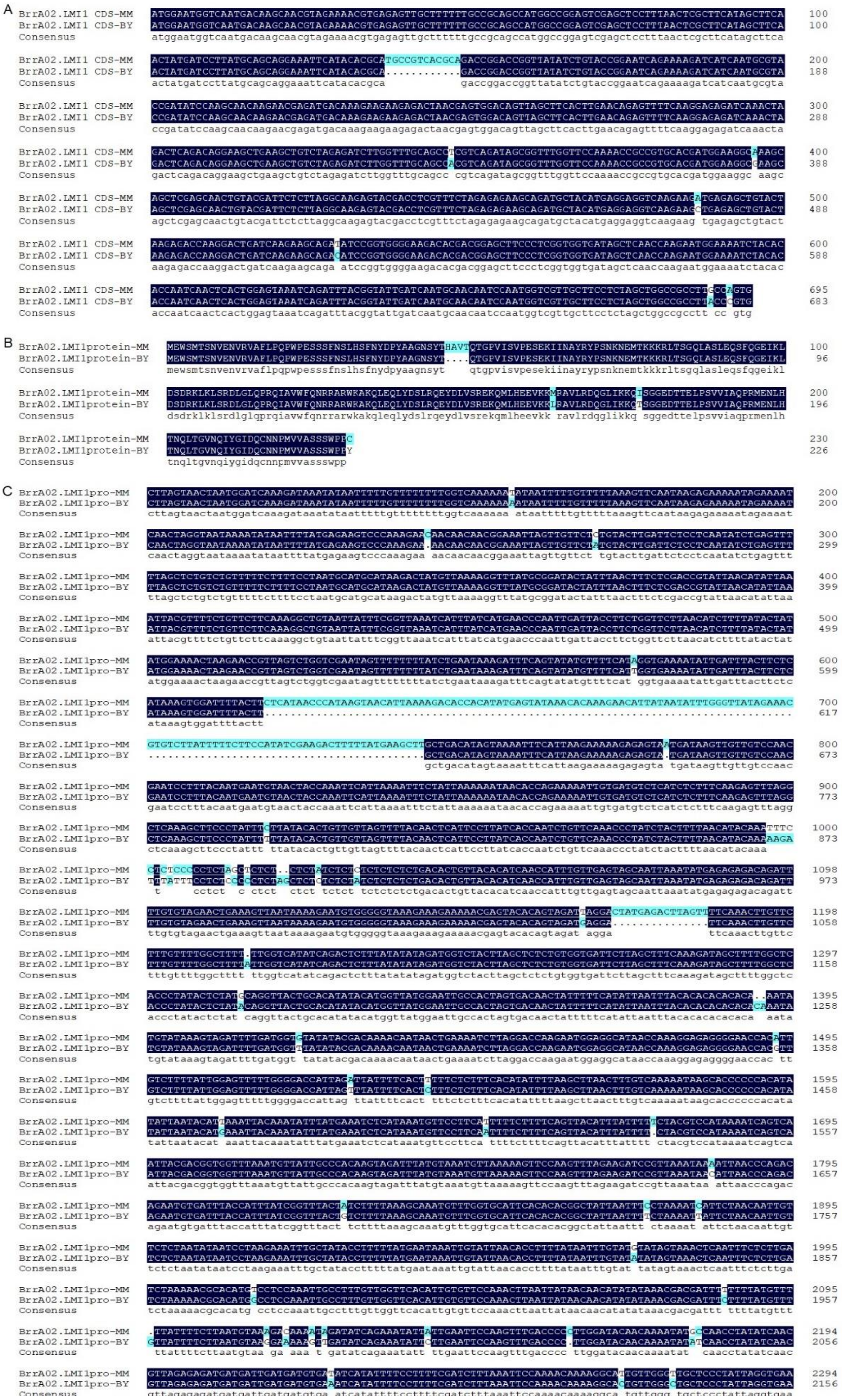

Figure S1 (A) Genomic sequence, (B) amino acid sequence and (C) promoter sequence alignment of *BrrA02.LMI1* in the MM and BY genomes.

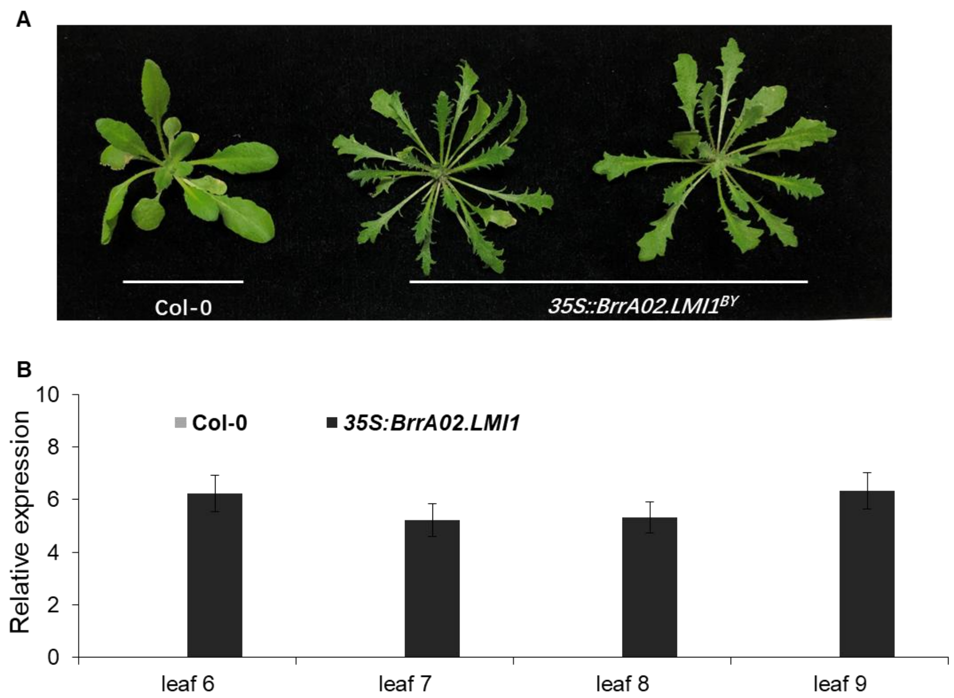

Figure. S2 (A) Leaf lobe phenotypes of independent T_2 *BrrA02.LMI1*^{BY}-overexpressing transgenic and wild-type *Arabidopsis*. Scale bars: 1 cm; (B) Expression level of *BrrA02.LMI1* in rosette leaves 6 to 9 of wild-type and *BrLMI1*^{BY}-overexpressing plants. Error bars represent standard errors derived from three replicates.

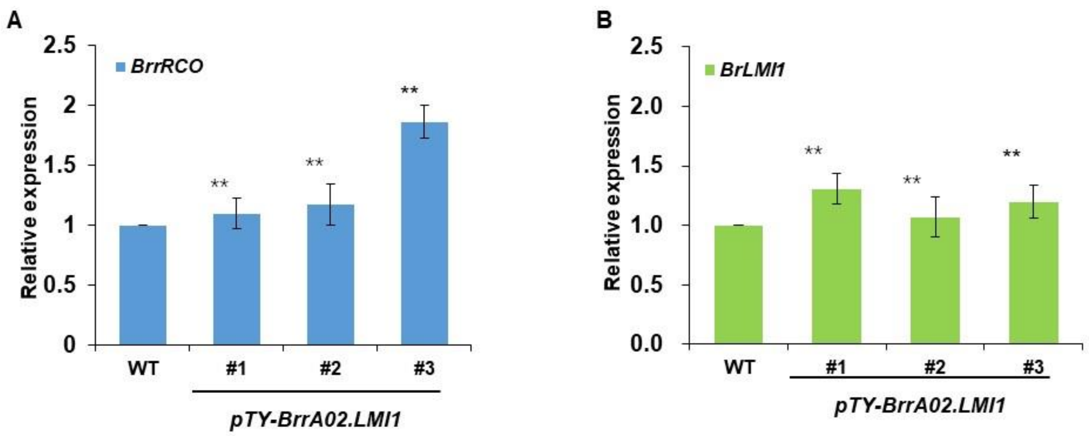

Figure. S3 The expression levels of two *BrrA02.LMI1* homologs in *B. rapa*, *BrLMI1* and *BrrRCO* in wild-type and *BrrA02.LMI1*-silenced plants. Values are the mean ± SD of three biological replicates.

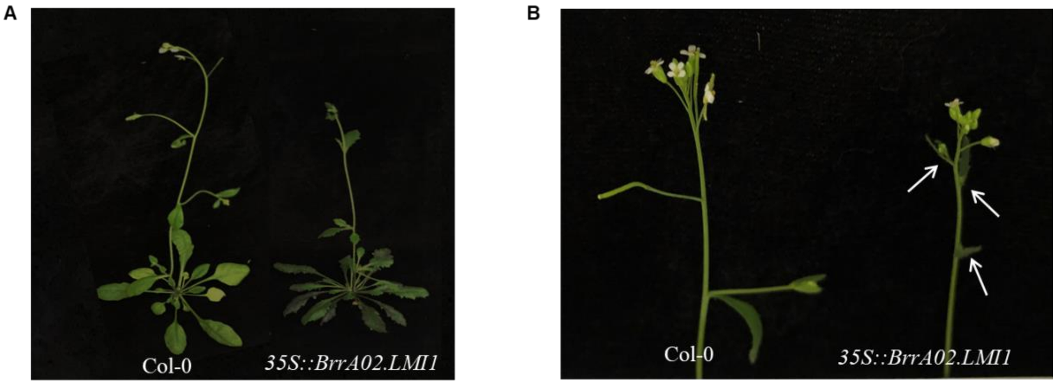

Figure. S4 *BrrA02.LMI1* showed pleiotropic effects on the development of plant organs. *BrrA02.LMI1* overexpressing transgenic lines exhibited enhanced lobed leaves and increased numbers of rosette leaves before bolting (A) and more bracts (B) in the reproductive stage.
